# Functional correlation of *ATP1A2* mutations with phenotypic spectrum: from pure hemiplegic migraine to its variant forms

**DOI:** 10.1186/s10194-021-01309-4

**Published:** 2021-08-12

**Authors:** Yingji Li, Wenjing Tang, Li Kang, Shanshan Kong, Zhao Dong, Dengfa Zhao, Ruozhuo Liu, Shengyuan Yu

**Affiliations:** 1 Department of Neurology, The First Medical Center of Chinese PLA General Hospital, Fuxing Road 28, Haidian District, 100853 Beijing, China; 2 School of Medicine, Nankai University, 300071 Tianjin, China

**Keywords:** Familial hemiplegic migraine, *ATP1A2*, Na^+^/K^+^-ATPase, Patch clamp

## Abstract

**Background:**

Mutations in *ATP1A2*, the gene encoding the α2 subunit of Na^+^/K^+^-ATPase, are the main cause of familial hemiplegic migraine type 2 (FHM2). The clinical presentation of FHM2 with mutations in the same gene varies from pure FHM to severe forms with epilepsy and intellectual disability, but the correlation of these symptoms with different *ATP1A2* mutations is still unclear.

**Methods:**

Ten *ATP1A2* missense mutations were selected according to different phenotypes of FHM patients. They caused pure FHM (FHM: R65W, R202Q, R593W, G762S), FHM with epilepsy (FHME: R548C, E825K, R938P), or FHM with epilepsy and intellectual disability (FHMEI: T378N, G615R, D718N). After ouabain resistance and fluorescence modification, plasmids carrying those mutations were transiently transfected into HEK293T and HeLa cells. The biochemical functions were studied including cell survival assays, membrane protein extraction, western blotting, and Na^+^/K^+^-ATPase activity tests. The electrophysiological functions of G762S, R938P, and G615R mutations were investigated in HEK293T cells using whole-cell patch-clamp. Homology modeling was performed to determine the locational distribution of *ATP1A2* mutations.

**Results:**

Compared with wild-type pumps, all mutations showed a similar level of protein expression and decreased cell viability in the presence of 1 µM ouabain, and there was no significant difference among the mutant groups. The changes in Na^+^/K^+^-ATPase activity were correlated with the severity of FHM phenotypes. In the presence of 100 µM ouabain, the Na^+^/K^+^-ATPase activity was FHM > FHME > FHMEI. The ouabain-sensitive Na^+^/K^+^-ATPase activity of each mutant was significantly lower than that of the wild-type protein, and there was no significant difference among all mutant groups. Whole-cell voltage-clamp recordings in HEK293T cells showed that the ouabain-sensitive pump currents of G615R were significantly reduced, while those of G762S and R938P were comparable to those of the wild-type strain.

**Conclusions:**

*ATP1A2* mutations cause phenotypes ranging from pure FHM to FHM with epilepsy and intellectual disability due to varying degrees of deficits in biochemical and electrophysiological properties of Na^+^/K^+^-ATPase. Mutations associated with intellectual disability presented with severe impairment of Na^+^/K^+^-ATPase. Whether epilepsy is accompanied, or the type of epilepsy did not seem to affect the degree of impairment of pump function.

**Supplementary Information:**

The online version contains supplementary material available at 10.1186/s10194-021-01309-4.

## Background

Hemiplegic migraine (HM) is characterized by severe migraine attacks with aura symptoms, including reversible motor weakness, usually affecting one side of the body and accompanied by visual, sensory, or language symptoms [1]. In some cases, patients experience additional neurological phenomena, such as coma [2–4], ataxia [5, 6], and seizures [7–10]. Individuals usually fully recover between episodes, but some patients develop permanent neurological manifestations involving cerebellar signs [11, 12] and intellectual disability [8, 13, 14]. Familial hemiplegic migraine (FHM) is diagnosed when at least one first- or second-degree relative also suffers HM attacks [1]. To date, mutations in the *CACNA1A*, *ATP1A2*, and *SCN1A* genes have been identified in FHM, and the corresponding forms are referred to as FHM1, FHM2, and FHM3, respectively [1]. In particular, more than ninety mutations were reported in *ATP1A2*, and two-thirds of HMs were familial and dominantly inherited [15]. The clinical presentation of FHM2 with mutations in the same gene varies from pure FHM to severe variant forms with epilepsy and intellectual disability, but the correlation of these symptoms with different *ATP1A2* mutations is still unclear.

The *ATP1A2* gene encodes the α2 subunit of Na^+^/K^+^-ATPase, which is a membrane-bound protein belonging to the P-type ATPase family [16]. Four different α isoforms are expressed in cells from different tissues [17]. *ATP1A2* is predominantly expressed in astrocytes of the adult brain [18], while *ATP1A3* is neuron-specific and mainly associated with alternating hemiplegia of childhood (AHC). It has been established that in addition to FHM2, mutations in *ATP1A2* can also lead to AHC [12, 19] accompanied by mental retardation with or without epilepsy, suggesting a clinical overlap with FHM. Studies have shown that *ATP1A3* mutations can be grouped by disease severity [20–22], but the clinical phenotypic spectrum has not been systematically studied for *ATP1A2* mutations.

Previously, we reported a novel *ATP1A2* mutation, G762S, that caused pure familial hemiplegic migraine [23], which inspired us to study the functional properties of FHM with different neurological symptoms. Therefore, this study aimed to systematically assess the phenotypic spectrum of previously reported *ATP1A2* mutations, investigate the functional impairment of ten mutations in the cytoplasmic domain that have not been fully characterized and correlate FHM2 phenotypes with the specific functional effects of mutations to better understand the characteristics of *ATP1A2* mutations, and to provide new perspectives for the pathogenesis, diagnosis, and treatment of similar diseases.

## Materials and methods

### cDNA constructs

Full-length human *ATP1A2* (NM_000702) and *ATP1B1* (NM_001677) cDNAs were purchased from OriGene (pCMV6-AT1A2-Entry, Cat#RC208606; pCMV6-AT1B1-Entry, Cat#RC200500, OriGene Technologies, Inc., Rockville, USA) and subcloned into the vectors pEF1α-IRES-AcGFP1 and pEF1α-IRES-DsRed-Express2, respectively. The wild type (WT) was designed containing p.Q116R and p.N127D to reduce ouabain sensitivity. Ten of previously identified variants were introduced to *ATP1A2* cDNA: R65W (c.193 C > T), R202Q (c.605G > A), T378N (c.1133 C > A), R548C (c.1642 C > T), R593W (c.1777 C > T), G615R (c.1843G > A), D718N (c.2152G > A), G762S (c.2284G > A), E825K (c.2473G > A), and R938P (c.2813G > C). Site-directed mutagenesis was carried out by PCR with specific primers using Invitrogen™ Platinum™ SuperFi™ DNA Polymerase (Cat#12,351,050, Invitrogen, Thermo Fisher Scientific, USA) as we previously described [23]. The recombinant fragments were amplified with PCR. The cDNA concentrations were determined by a microvolume spectrophotometer (Pultton P100/P100+, San Jose, CA, USA). Whole-insert sequencing was performed to verify the DNA sequence identity.

### HeLa cells and HEK293T cells

HeLa cells and HEK293T cells were kindly provided by Dr. Weidong Han (Department of Bio-Therapeutic, Chinese PLA General Hospital, Beijing, China) and cultured with high-glucose DMEM (Dulbecco’s modified Eagle’s medium) containing 1 % penicillin-streptomycin and 10 % fetal bovine serum in 5 % CO_2_ at 37 °C. Cells within 20 passages were used for transient transfection using Lipofectamine 3000 (Cat#L3000015, Invitrogen, Carlsbad, CA, USA) in reduced-serum Opti-MEM. HEK293T cells were grown in 6-well dishes and transfected with *ATP1A2* (WT and/or mutant constructs) and *ATP1B1* at a ratio of 2:1. After 48 h of transfection, cells were reseeded on poly-L-lysine-coated glass slips for electrophysiological recordings.

### Transient transfection and cell survival assay

HeLa and HEK293T cells were seeded in 96-well dishes at 10,000 cells per well. Twenty-four hours after plating, cells were transfected with cDNA constructs of 0.1 µg per well. After 24 h of transfection, 1 µM ouabain was added to the medium, which can inhibit endogenous Na^+^/K^+^-ATPase without affecting ouabain-resistant pumps [24]. Cell viability was assessed at 12 h, 24 h, 36 h, 48 h, 60 h, and 72 h after exposure to ouabain using a methyl thiazolyl tetrazolium (MTT) reduction assay kit (BB-4201, BestBio, Shanghai, China). Another set of experiments tested the cell survival rate in the absence of ouabain. Formazan salt formation was monitored at 492 nm using a Multiskan FC Microplate Photometer (Thermo Scientific, USA). All experiments were carried out at least 3 times, with each data point performed in quintuplicate. The survival rate of the transfected groups was calculated relative to that of nontransfected controls.

### Isolation of membrane fractions and western blotting

Total and plasma membranes from HeLa cells were isolated using a Minute™ Plasma Membrane Protein Isolation and Cell Fractionation Kit (Cat#SM-005, Invent Biotechnologies, Inc., Plymouth, USA). After 48 h of transfection, approximately 5 × 10^7^ cells were collected and sensitized by Buffer A. The ruptured cell membranes and intact nuclei were obtained by passing through the proprietary filter twice with centrifuging at 16,000×g for 30 s. The total membranes were collected by centrifuging the resuspended pellet at 700×g for 1 min and subsequently centrifuging the supernatant for 30 min at 16,000×g. For isolation of plasma membranes, the total membrane pellet was resuspended in Buffer B and centrifuged at 7800×g for 5 min. The supernatant was transferred to a fresh tube with 1.6 ml cold PBS and centrifuged at 16,000×g for 30 min.

The whole steps were performed at 4℃. All buffers included 1 mM phenylmethylsulfonyl fluoride (PMSF) and cOmplete ULTRA protease inhibitor cocktail tablets (Cat#05892970001, Roche Diagnostics GmbH, Mannheim, Germany). The pelleted membranes were stored at -70 °C and resuspended in Minute™ Denaturing Protein Solubilization Reagent (Cat#WA-009, Invent Biotechnologies, Inc., USA) before western blotting. A bicinchoninic acid (BCA) protein assay kit (Cat#E162-01, GenStar, Beijing, China) was used to determine the protein concentration.

Approximately 10 µg of solubilized protein sample was mixed with SDS-PAGE loading buffer, separated on an SDS gel containing 8 % acrylamide and blotted on a polyvinylidene difluoride membrane. The α2 subunit of Na^+^/K^+^-ATPase was detected by rabbit monoclonal antibody [EPR11896(B)] (dilution 1:1000, ab166888, Abcam, Cambridge, UK), and membranes were incubated overnight at 4 °C. Horseradish peroxidase-labeled goat anti-rabbit IgG (H + L) (1:750, Cat#A0208, Beyotime Biotechnology, Shanghai, China) was used as a secondary antibody. An α-tubulin rabbit polyclonal antibody (1:1000, Cat#AF0001, Beyotime Biotechnology, Shanghai, China) was used as a protein loading control. ECL chemiluminescence reagent (Cat#SL1350, Coolaber Science & Technology Co., Beijing, China) was used for imaging. All experiments were carried out at least three times in two separate clones. The relative intensity of western blotting was analyzed using ImageJ.

### Na^+^/K^+^-ATPase activity test

ATPase can decompose ATP to generate ADP and Pi (inorganic phosphorus). The activity of ATPase can be evaluated by measuring the amount of Pi. HeLa cells were harvested after 48 h of transfection and washed with cold saline to make a 10^7^/ml cell suspension. Then, a homogenizer was used to disrupt the cells. The BCA protein assay kit (Cat#E162-01, GenStar, Beijing, China) was used to determine the protein concentration. Na^+^/K^+^-ATPase activity was determined following the manufacturer’s instructions for the Ultra Trace Sodium Potassium ATPase Test Kit (Cat#A070-2, Nanjing Jiancheng Bioengineering Institute, China). Approximately 100 µl membranes were combined with reagents A, B and C and incubated at 37 °C for 30 min, after which reagent D was added to terminate the enzymatic reaction. The mixture was centrifuged at 3500 rpm for 10 min. The supernatant was then extracted for measurement of the phosphorus concentration. After adding the developer and maintaining at room temperature for 5 min, the absorbance of each tube was measured at 636 nm. Ouabain (10 µM or 100 µM) was added to inhibit endogenous ATPase. Ouabain-sensitive ATPase activity was determined by subtracting the amount of Pi formed in the presence of 10 µM and 100 µM ouabain. All experiments were performed in triplicate in at least four separate clones.

### HEK293T whole-cell patch clamp

Electrophysiological recordings were made at room temperature (22–24℃) using an EPC 10 USB amplifier (HEKA Elektronik GmbH, Lambrecht/Pfalz, Germany). Patch pipettes were pulled by a MODEL P-97 Flaming/Brown Micropipette Puller (Sutter Instrument, Novato, USA) using borosilicate glass capillaries (Item#: BF150-86-10, Sutter Instrument, Novato, CA, USA). The pump current was recorded with an electrode resistance of 3–4 MΩ in buffer containing (in mM) 95 NaMeSO_4_, 20 TEA-Cl, 2 BaCl_2_, 5 EGTA, 4 Mg-ATP, 5 Tris-phosphocreatine, 10 HEPES, 5 glucose, pH adjusted to 7.35 with NaOH, 290 mOsm. The extracellular solution contained (in mM): 140 NaCl, 1 MgCl_2_, 2 BaCl_2_, 2 CsOH, 2 NiCl_2_, 0.2 CdCl_2_, 0.33 NaH_2_PO_4_, 5 HEPES, 5 glucose, 20 NMDG-Cl, pH adjusted to 7.35 with HCl, and 320 mOsm [25]. All recording solutions included 1 µM ouabain to block endogenous pumps. Cells were continuously held at -30 mV or + 40 mV and perfused with a K^+^-free solution. The pump current was activated by superfusing cells with an extracellular solution containing 20 mM KCl instead of 20 mM NMDG-Cl. For the analysis of voltage dependence, currents were elicited with 200 ms test steps from − 100 to + 100 mV, increased by + 20 mV, and at a -70 mV holding potential. Then, 10 µM ouabain was added to measure the ouabain-sensitive current. Recordings were sampled at 50 kHz and filtered at 5 kHz.

### Homology modeling

We performed homology modeling in SWISS-MODEL using the sequence of human α2 isomer (UniProt IDs P50993) based on crystal structures of the Na^+^/K^+^-ATPase from the bovine kidney (Protein Data Bank accession number 4XE5 recently solved at 3.9 Å resolution) [26], with which it shares 86.95 % sequence identity. Three-dimensional models of the human Na^+^/K^+^- ATPase were analyzed by the PyMOL(TM) Molecular Graphics System (Version 2.3.0).

### Statistical analysis

Electrophysiological data were analyzed using FitMaster v2 × 90.2 (HEKA Elektronik GmbH, Lambrecht/Pfalz, Germany). Statistics and plotting were performed using R software 3.6 (R Foundation for Statistical Computing, Vienna, Austria). Statistical analysis was performed with one-way ANOVA (Tukey HSD test for multiple comparisons) or Kruskal-Wallis tests. The current-voltage curves (or MTT cell survival assays) were compared with a two-way ANOVA. Linear regression was used to calculate the correlation between the age of first HM onset and Na^+^/K^+^-ATPase activity. Data are presented as the mean ± standard error (SE) with a significance level of *P* < 0.05.

## Results

### *ATP1A2* mutations and clinical phenotypic spectrum

**Fig. 1:**
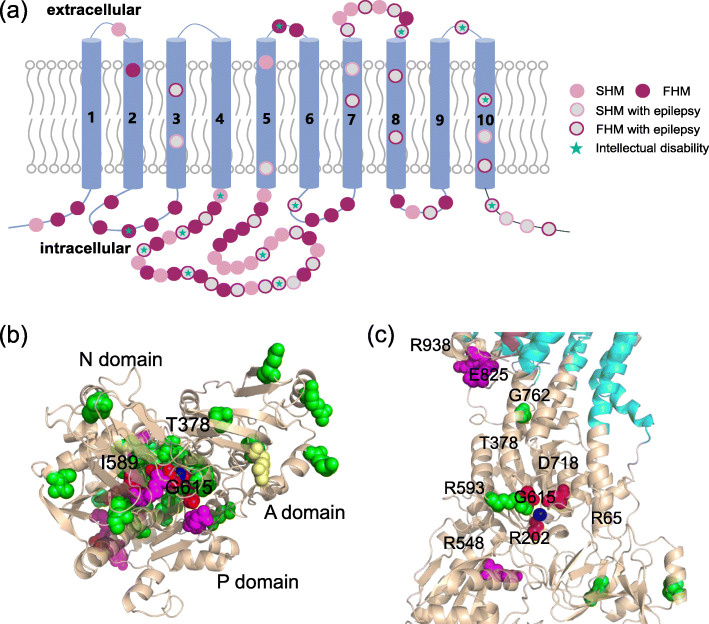
Structural location of *ATP1A2* mutations.** a** Phenotypic distribution of *ATP1A2* missense mutations (colored circles, *n* = 83). Ten transmembrane segments are shown as blue cylinders. **b** *ATP1A2* mutant residues are shown as spheres and divided into four groups: pure HM (green), HM with epilepsy (magenta), HM with ataxia (yellow) and HM with intellectual disability (with or without epilepsy) (red) according to the clinical symptoms of patients. The intracellular loops are shown in the top view, indicating that mutations causing severe HM (accompanied by intellectual disability) were closer to Mg^2+^ (blue) near the phosphorylation site. **c** The structural locations and phenotypes of the ten mutations investigated in this study

All missense mutations are shown in the structural schematic diagram of the α2 subunit (Fig. 1a), of which 67.5 % were familial and 32.5 % were sporadic. Phenotypes associated with epilepsy and intellectual disability were mostly reported in familial cases, accounting for 48.2 and 21.4 %, respectively. The crystal structure of Na^+^/K^+^-ATPase from the bovine kidney (4XE5) was used as a template for homology modeling. *ATP1A2* missense mutations were marked by the associated clinical symptoms: pure HM, HM with epilepsy, HM with ataxia, and HM with intellectual disability (with or without epilepsy). From the top view of the intracellular loops (Fig. 1b), we found that mutations causing HM with intellectual disability were closer to the phosphorylation site. Ten mutations at the cytoplasmic domain were studied to explore the relationships between phenotypic spectrum and function (Fig. 1c), which were from the literature search and our previous reports (Supplementary Table 1). Of these mutations, four cause pure FHM (R65W, R202Q, R593W, and G762S), three cause FHM with epilepsy (R548C, E825K, and R938P), and three cause FHM/AHC with epilepsy and intellectual disability (G615R, D718N, and T378N). The detailed sources and pedigrees of the studied FHM families are included in the Supplementary Material.

### *ATP1A2* expression remained unchanged between the mutant and wild type

**Fig. 2:**
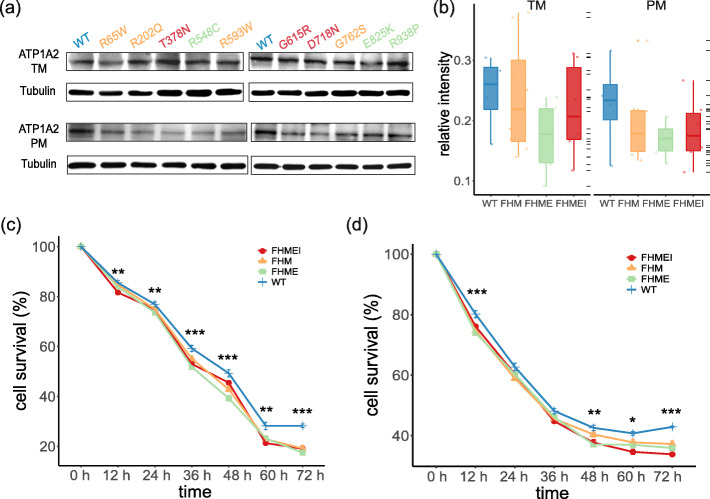
Expression and cell viability of mutant Na^+^/K^+^-ATPase.** a **Western blot of total membrane and plasma membrane proteins expressed in transfected HeLa cells (ATP1A2: 102 kDa; Tubulin: 50 kDa). The colored labels represented different groups: blue, WT; yellow, FHM; green, FHME; red, FHMEI. **b** The *ATP1A2* expression of mutants was equal to that of the WT (compared with WT, TM: *P* = 0.3822, PM: *P* = 0.5507, Kruskal-Wallis test). **c and d** Cell survival assays. All mutants showed a significantly reduced survival rate under 1 µM ouabain in both HEK293T (**c**) and HeLa (**d**) cells (*P* < 0.0001, two-way ANOVA)

*ATP1A2* mutations with distinct phenotypes were grouped as pure FHM (FHM), FHM with epilepsy (FHME), and FHM with epilepsy and intellectual disability (FHMEI). We found that *ATP1A2* mutations did not affect the levels of either total membrane proteins or plasma membrane proteins. The relative intensities of western blot analysis (Fig. 2a and b) showed a slight reduction in the mutant plasma membrane protein compared to WT that was not statistically significant (*P* > 0.05, Kruskal-Wallis test).

### *ATP1A2* mutations reduced cell survival under ouabain

The transfection efficiency, as estimated by the fluorescence intensity, showed no significant differences between the mutants and WT (Supplementary Fig. 1). We found a significantly reduced survival rate of mutants compared with WT in all three groups (*P* < 0.0001, two-way ANOVA, Fig. 2c and d), indicating that the mutant pump significantly affected the function of the Na^+^/K^+^-ATPase. The individual mutants also showed a reduced survival rate compared with that of WT pumps in HeLa cells (R65W: *P* = 0.032, other mutants: *P* < 0.0001, two-way ANOVA) and HEK293T cells (R65W: *P* = 0.016, other mutants: *P* < 0.0001, two-way ANOVA) (Supplementary Figs. 2 and 3). In the absence of ouabain, the survival rates of cells in each mutant group were similar to those in the WT group (Supplementary Fig. 4).

### The Na^+^/K^+^-ATPase activity of the *ATP1A2* mutant was associated with the phenotypic spectrum

**Fig. 3:**
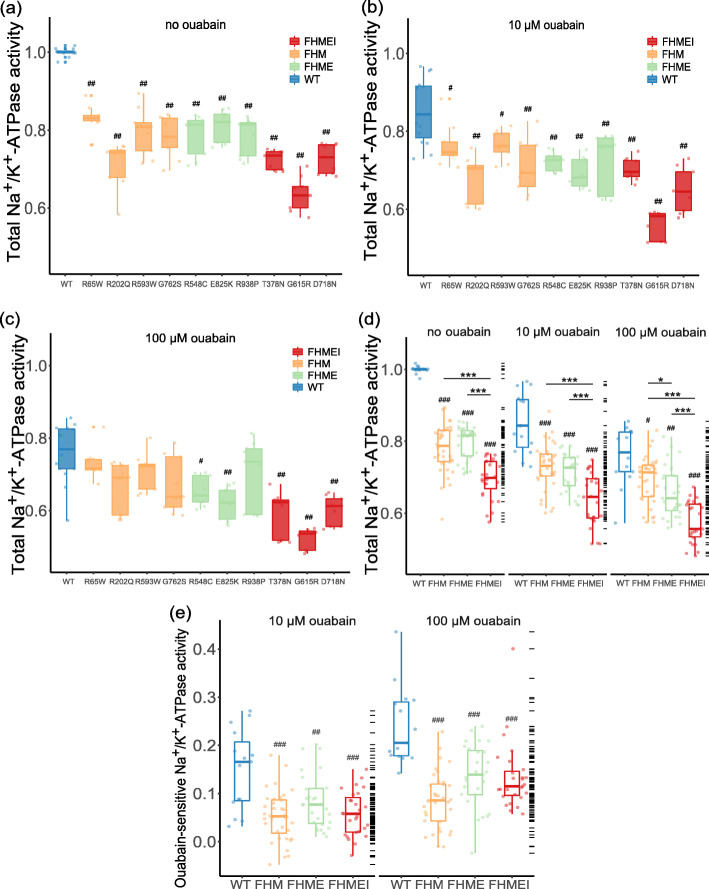
Na^+^/K^+^-ATPase activity test.** a-c** Na^+^/K^+^-ATPase activity of each mutant in the absence and presence of 10 µM or 100 µM ouabain. The activity of the Na^+^/K^+^-ATPase was normalized to that of the WT Na^+^/K^+^-ATPase without ouabain. **d** Na^+^/K^+^-ATPase activity with and without ouabain. Mutations were divided into three groups according to their FHM phenotypes. **e** The ouabain-sensitive ATPase activity of the WT pump was higher than that of mutant pumps in the presence of 10 µM and 100 µM ouabain. *P* value compared with the WT: ^#^*P* < 0.05, ^##^*P* < 0.01, ^###^*P* < 0.001, Kruskal-Wallis test. *P* value between groups: *P < 0.05, **P < 0.01, ***P < 0.001, Kruskal-Wallis test

We found that the Na^+^/K^+^-ATPase activity of each mutant was lower than that of the WT in the absence of ouabain (Fig. 3a, *P* < 0.01), and similar results were obtained in the presence of 10 µM ouabain (Fig. 3b, *P* < 0.05). In the presence of 100 µM ouabain, compared with WT, only the R548C, E825K, T378N, G615R and D718N mutations showed reduced Na^+^/K^+^-ATPase activity (*P* < 0.05), while R65W, R2020Q, R593W, G762S and R938P showed Na^+^/K^+^-ATPase activity similar to that of WT protein (Fig. 3c, *P* > 0.05). Of note, the Na^+^/K^+^-ATPase activity of WT was decreased under 10 µM and 100 µM ouabain compared with that in the absence of ouabain (*P* < 0.0001). When mutations were divided according to their FHM phenotypes, we found that in the absence and presence of 10 µM ouabain, the Na^+^/K^+^-ATPase activity of the WT was higher than that of the mutants in all phenotype groups (Fig. 3d compared with WT, *P* < 0.0001). Of interest, the overall Na^+^/K^+^-ATPase activity of mutations causing FHMEI was significantly lower than that of mutations causing FHM (*P* < 0.0001) and FHME (*P* = 0.0003) in the presence of 10 µM ouabain. However, there was no significant difference between FHM and FHME mutations (*P* = 0.294). In the presence of 100 µM ouabain, the level of Na^+^/K^+^-ATPase activity was as follows: WT > FHM > FHME > FHMEI. Specifically, the Na^+^/K^+^-ATPase activity of WT was significantly higher than that of mutants associated with FHM (*P* = 0.012) and FHME (*P* = 0.002) and FHMEI (*P* < 0.0001); the Na^+^/K^+^-ATPase activity of FHM mutants was significantly higher than that of FHME (*P* = 0.040) and FHMEI (*P* < 0.0001) mutants; and the Na^+^/K^+^-ATPase activity of FHME mutants was significantly higher than that of FHMEI mutants (*P* = 0.0003).

We further analyzed the ouabain-sensitive Na^+^/K^+^-ATPase activity in the presence of 10 µM and 100 µM ouabain (Fig. 3e). The ouabian-sensitive Na^+^/K^+^-ATPase activity was calculated by subtracting the total Na^+^/K^+^-ATPase activity obtained under different concentrations of ouabain from that obtained without ouabain. Consistent with the above findings, the ouabain-sensitive Na^+^/K^+^-ATPase activity of WT was significantly higher than that of the mutant groups at 10 µM ouabain (compared with WT: FHME, *P* = 0.002, FHM and FHMEI, *P* < 0.0001), while no significant difference was found among mutant groups (*P* > 0.05). Similarly, in the presence of 100 µM ouabain, the Na^+^/K^+^-ATPase activity of the WT group was significantly higher than that of the mutant group (*P* < 0.0001).

**Fig. 4:**
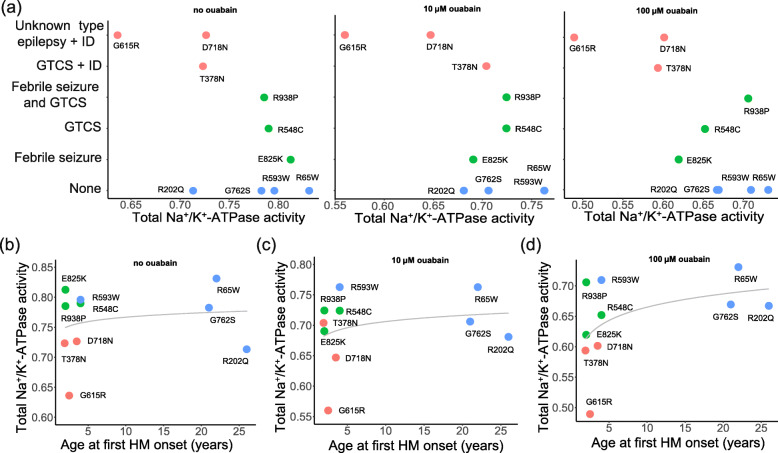
Phenotypic effects on total Na^+^/K^+^-ATPase activity. The phenotype of the *ATP1A*2 mutations: red, FHM/AHC with epilepsy and intellectual disability; green, FHM with epilepsy; blue, pure FHM. **a** The activity of mutant Na^+^/K^+^-ATPase associated with intellectual disability showed lower activity than that of pure FHM or with epilepsy. GTCS, generalized tonic-clonic seizure; ID, intellectual disability. **b-d** Among mutations associated with epilepsy and intellectual disability, patients had a younger age of first hemiplegic migraine attack, but there was no statistical correlation with Na^+^/K^+^-ATPase activity (no ouabain: *P* = 0.597, 10 µM ouabain: *P* = 0.463, 100 µM ouabain: *P* = 0.169). HM, hemiplegic migraine

We drew a scatter plot based on the phenotypic information in Supplementary Table 1 (Fig. 4a). Consistent with our above results, the mutant Na^+^/K^+^-ATPase activity associated with intellectual disability was low, and whether epilepsy was accompanied, or the type of epilepsy did not seem to have an effect on Na^+^/K^+^-ATPase activity. We also noticed that patients with epilepsy and intellectual disability were younger at first HM attack, while patients with pure FHM were older at first attack. The regression analysis was conducted between age at first HM attack and Na^+^/K^+^-ATPase activity, but no statistical significance was found (Fig. 4b-d, *P* > 0.05).

**Fig. 5:**
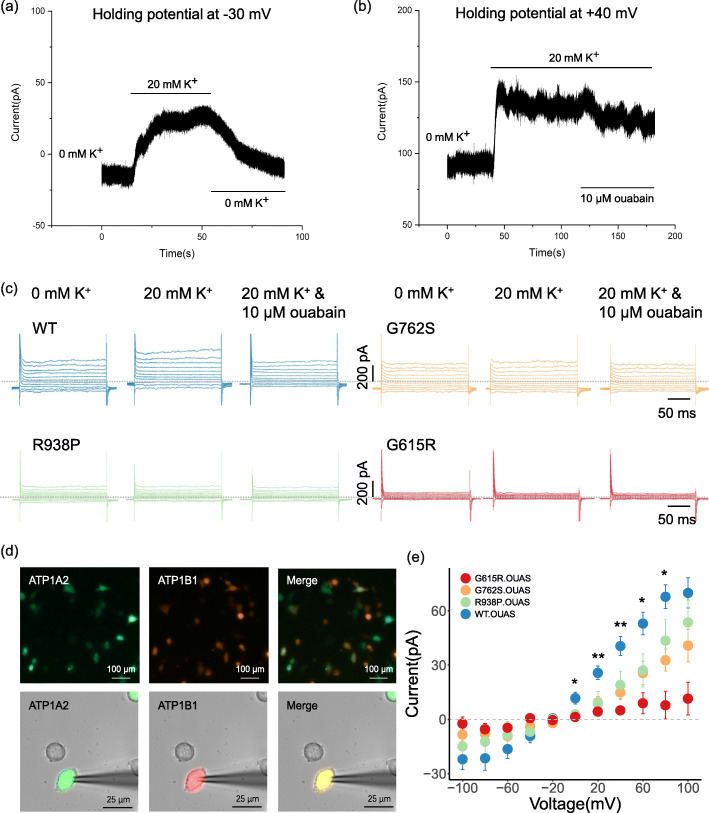
Pump current recordings of Na^+^/K^+^-ATPase transfected in HEK293T cells.** a and b** Continuous recordings with wild-type pumps in HEK293T cells. 1 µM ouabain was added to block endogenous pumps. The pump current was activated by superfusion of 20 mM K^+^ and inhibited by 10 µM ouabain. **c** Representative raw currents of WT (blue), G762S (yellow), R938P (green) and G615R (red) in response to 200 ms steps with + 20 mV increments from − 100 to + 100 mV. **d** Coexpression of *ATP1A2* (green) and *ATP1B1* (red) in transfected HEK293T cells. **e** Ouabain-sensitive pump currents measured in the last 50 ms of test pulses (two-way ANOVA: *P* = 0.040) (amplitude at + 40 mV, one-way ANOVA compared with WT, *n* = 9; G762S, *n* = 10, *P* = 0.018; R938P, *n* = 8, *P* = 0.142; G615R, *n* = 10, *P* = 0.008)

### The mutation causing a severe phenotype showed lower pump currents

HEK293T cells were continuously held at -30 mV or + 40 mV to record pump currents in the WT pump (Fig. 5a and b). The pump current was activated by superfusion of 20 mM K^+^ and inhibited by 10 µM ouabain (Fig. 5c). Cells with co-fluorescence expression were selected for electrophysiological recording (Fig. 5d). We found that the ouabain-sensitive pump current in the mutant was slightly reduced for G762S and R938P compared to the WT Na^+^/K^+^-ATPase (Fig. 5e, G762S, *P* = 0.185, R938P, *P* = 0.443, two-way ANOVA) with no statistically significant difference. In G615R, the ouabain-sensitive pump currents were significantly reduced and close to zero (Fig. 5e, *P* = 0.042, two-way ANOVA). However, at + 40 mV, the ouabain-sensitive pump currents of G762S (14.91 ± 4.12 pA) and G615R (4.98 ± 1.61 pA) were significantly decreased compared to those of WT (40.56 ± 5.20 pA, *P* < 0.05), and P938P (19.06 ± 13.48 pA) showed currents comparable to those of WT (*P* > 0.05).

## Discussion

Since the first report associated mutations of the Na^+^/K^+^-ATPase α subunit with heritable disease [9], the phenotypic spectrum of mutations in this gene has been expanding considerably. Most *ATP1A2* mutations cause familial or sporadic hemiplegic migraine, some accompanied by ataxia [5], a range of epilepsies (from benign familial infantile convulsions to generalized epilepsy with febrile seizures plus [7, 10]) or intellectual disability [4, 27]. In this study, we compared the effect of *ATP1A2* mutations covering this phenotypic spectrum of FHM2 to understand the underlying mechanisms and to correlate genotypes with phenotypes.

### Functional consequences of *ATP1A2* mutations

Our results suggested that all mutant constructs expressed a level of protein (total membrane or plasma membrane) similar to that of the WT pump. This indicated that *ATP1A2* mutants did not affect the synthesis or targeting of Na^+^/K^+^-ATPase. In vertebrates, the β-subunit acts as an important ancillary subunit of Na^+^/K^+^-ATPase, which mainly assists in correct protein folding, membrane insertion, and plasma membrane delivery [17]. In this study, the ten included mutations were all located in the intracellular domain, so there was little interaction of these residues with the β subunit. Of note, both the expression vector used and the culture temperature have important effects on protein expression and function [28], which makes it difficult to reach consistent conclusions in comparison to previous studies. Most *ATP1A2* mutations showed protein levels similar to those of the WT [29, 30]. Mutations associated with reduced total or plasma membrane protein levels were either located in transmembrane helices, such as G301R [2] and G855R [31]; interacted with the β subunit directly or indirectly, such as W887R [32] and R908Q [28]; or were more severe mutation types, such as del(K935_S940)ins(Ile) [29], S996fs [29] and L994del [31]. This suggested that missense mutations in intracellular domains had little effect on membrane protein expression and that there may be other mechanisms that contribute to the pathogenicity of these mutations. Similar results were obtained in the study of *ATP1A3* [33], although AHC tends to be more severe than rapid-onset dystonia-parkinsonism, no difference in protein expression was found between groups. In this study, all mutations showed decreased cell survival under 1 µM ouabain compared with the WT, but there were no differences among the mutant groups. In fact, we found that almost all *ATP1A2* mutations reported in previous studies resulted in a significant decrease in cell viability, whether in HeLa cells or HEK293T cells. Although we speculated that mutations causing more severe accompanying symptoms might show significantly reduced plasma membrane levels and cell survival, it was difficult to correlate those functional damages with the severity of disease based on previous research and the current results.

At present, most assays of mutant Na^+^/K^+^-ATPase activity have been performed in SF9 insect cells [30, 34], while a few studies involving C515Y [35] and L764P [36] have used Xenopus oocytes. Most *ATP1A2* mutations showed significantly reduced ouabain-sensitive Na^+^/K^+^-ATPase activity, while only a few mutants, including Y9N, R51H, E174K, T345A, R897Q, and R879W [30, 34], showed activity similar to that of WT. The mutants with normal Na^+^/K^+^-ATPase activity were all associated with pure FHM, pure SHM or the more common migraine with or without aura, which suggested that ouabain-sensitive Na^+^/K^+^-ATPase activity may be related to the disease severity. Of interest, we found a correlation between the severity of the FHM phenotype and the total Na^+^/K^+^-ATPase activity rather than ouabain-sensitive Na^+^/K^+^-ATPase activity, which has not been considered in previous studies. In our experiments, ouabain was added to inhibit the endogenous Na^+^/K^+^-ATPase activity, so the ouabain-sensitive Na^+^/K^+^-ATPase activity here more represented the endogenous part. We believed that the total Na^+^/K^+^-ATPase activity represented the exogenous pump activity of transfection, and this part of the Na^+^/K^+^-ATPase activity was found to related to the severity of the disease.

Pump currents were recorded in homologous HEK293T cells. We found reduced ouabain-sensitive pump currents for the G615R mutant protein compared with the WT pump, while no difference was found for G762S or R938P. This result was consistent with our findings for Na^+^/K^+^-ATPase activity, indicating a correlation between the pump current and the severity of FHM neurological accompanying symptoms. In previous studies, variants showing reduced or undetectable pump currents mainly involved severe mutations such as L994del, del(K935_S940) ins(Ile), and S966fs [29]. In addition, mutations near the C-terminal ion pathway, D999H, Y1009X, and R937P [31], also presented decreases in pump current. This suggested that there may be a link among the structural location, FHM phenotype and functional impairment.

### Genotype-phenotype correlation and impact of *ATP1A2* pathogenic variants

For the first time, we studied the relationship of different phenotypes in *ATP1A2* mutations with functional impairment. All investigated mutations showed similar levels of protein expression to that of the WT and decreased cell viability, demonstrating that as a rough estimate of pump function, the membrane expression and cell survival assays cannot distinguish FHM phenotypes of different severities. We argued that amplitudes of Na^+^/K^+^-ATPase activity and pump currents are related to the FHM phenotypes, and whether it was accompanied by epilepsy, or the type of epilepsy had little effect on Na^+^/K^+^-ATPase activity. A recent review demonstrated that among the three monogene mutations causing FHM, most cases associated with epilepsy occurred in *ATP1A2* mutations [37]. However, the prognosis of epilepsies was benign [37]. In addition, our study found that functional changes of epilepsy-related mutations are similar to mutations associated with pure FHM, indicating that intellectual disability was an important indicator of severe functional impairment of the mutant pump. Severely decreased Na^+^/K^+^-ATPase activity and a reduction in pump currents were found in the G615R mutation causing FHM with epilepsy and intellectual disability. Two sporadic cases that were reported with a different amino acid change, G615E, showed HM with generalized tonic-clonic seizures, and another sporadic case carrying the G615E mutation was diagnosed with cognitive and motor delay at two years old, indicating the structural importance of G615 [4]. Of note, G615R was associated with an incomplete penetrance of 2/5 of intellectual disability and 4/5 of epilepsy in heritable cases. Thus, both the mutation itself and other genetic or environmental factors contribute to the individual phenotype.

Na^+^/K^+^-ATPase uses the energy derived by ATP hydrolysis to transport three Na^+^ out of the cell and two K^+^ into the cell [16]. Our results reveal that varying degrees of Na^+^/K^+^-ATPase loss-of-function exist in different FHM phenotypes, supporting a haploinsufficiency mechanism. It is expected to impair the reuptake of K^+^ and glutamate in the synaptic crevices, thus leading to hyperexcitability of the brain and increased susceptibility to cortical spreading depression (CSD), which is associated with migraine auras. Mutations in the ion transport genes *CACNA1A*, *ATP1A2* and *SCN1A* can lead to familial hemiplegic migraine, indicating genetic heterogeneity. Previous studies suggested that gain-of-function FHM1 mutations were associated with increased susceptibility to CSD. However, a recent study reported a *de novo CACNA1A* mutation causing hemiplegic migraine and developmental delay but without epilepsy, revealing a loss-of-function of Ca_v_2.1 channel [38]. This confirmed that different functional consequences may occur in patients with intellectual disability. Our study also demonstrated that severely decreased Na^+^/K^+^-ATPase activity and a reduction in pump currents were only found in mutations associated with intellectual disability. These findings suggest that hemiplegic migraine, epilepsy, and intellectual disability may have a common pathogenesis, that is, damage to sodium-potassium pump function. And different degrees of damage lead to different neurological manifestations. It is known that severe epilepsy is often accompanied by intellectual disability, but intellectual disability can also occur without seizures. Recent studies on sodium channels showed that mutations causing intellectual disability with or without epilepsy manifested as different effects on channel function and neuronal firing [39, 40]. Although our results suggest that mutations associated with intellectual disability manifest as more severe pump impairment, their role in glial cells and their relationship with neuronal firing are still unclear. More extensive neuron-related research should be conducted in the future, and the study of neuron networks and mouse models is essential for understanding the mechanism of nervous system disease.

## Conclusions

In conclusion, this study provides a comprehensive delineation of the phenotypic spectrum with *ATP1A2* mutations ranging from pure FHM to FHM with epilepsy and/or intellectual disability. We argue that *ATP1A2* mutations cause a variety of phenotypes that are well correlated with distinct consequences of the mutations on biochemical and biophysical properties. Mutations associated with intellectual disability often cause more serious functional impairment and need to be given more attention.

## Supplementary Information

**Additional file 1: Supplementary Figure 1.** Representative images of transfection efficiency of wild-type and mutant constructs with 100μm scales. **Supplementary Figure 2.** Survival assays in HeLa cells. The survival rate of each mutant was significantly reduced compared to the wild-type (R65W: *P* = 0.032, other mutants: *P* < 0.0001, two-way ANOVA). **Supplementary Figure 3.** Survival assays in HEK293T cells. The survival rate of each mutant was significantly reduced compared to the wild-type (R65W: *P* = 0.016, other mutants: *P* < 0.0001, two-way ANOVA). **Supplementary Figure 4. **Survival assays in HeLa cells in the absence of ouabain. The survival rates of cells in each mutant group were similar to those in the WT group (*P* > 0.05, two-way ANOVA). **Supplementary Table 1.** Clinical characteristics of previously published patients with studied ATP1A2 mutations.

## Data Availability

The datasets used and/or analyzed during the current study are available from the corresponding author on reasonable request.

## Abbreviations

FHM2: Familial hemiplegic migraine type 2
AHC: Alternating hemiplegia of childhood
WT: Wild type
CSD: Cortical spreading depression
