# Supplementary material for: Functional correlation of ATP1A2 mutations with phenotypic spectrum: from pure hemiplegic migraine to its variant forms
Source: J Headache Pain. 2021 Aug 12;22(1):92. doi: 10.1186/s10194-021-01309-4 (PMC8359390; doi:10.1186/s10194-021-01309-4)
Supplement: Supplementary file 1 — Additional file 1: Supplementary Figure 1. Representative images of transfection efficiency of wild-type and mutant constructs with 100μm scales. Supplementary Figure 2. Survival assays in HeLa cells. The survival rate of each mutant was significantly reduced compared to the wild-type (R65W: P = 0.032, other mutants: P < 0.0001, two-way ANOVA). Supplementary Figure 3. Survival assays in HEK293T cells. The survival rate of each mutant was significantly reduced compared to the wild-type (R65W: P = 0.016, other mutants: P < 0.0001, two-way ANOVA). Supplementary Figure 4. Survival assays in HeLa cells in the absence of ouabain. The survival rates of cells in each mutant group were similar to those in the WT group (P > 0.05, two-way ANOVA). Supplementary Table 1. Clinical characteristics of previously published patients with studied ATP1A2 mutations. [file 10194_2021_1309_MOESM1_ESM.docx]

**Supplementary Figures**


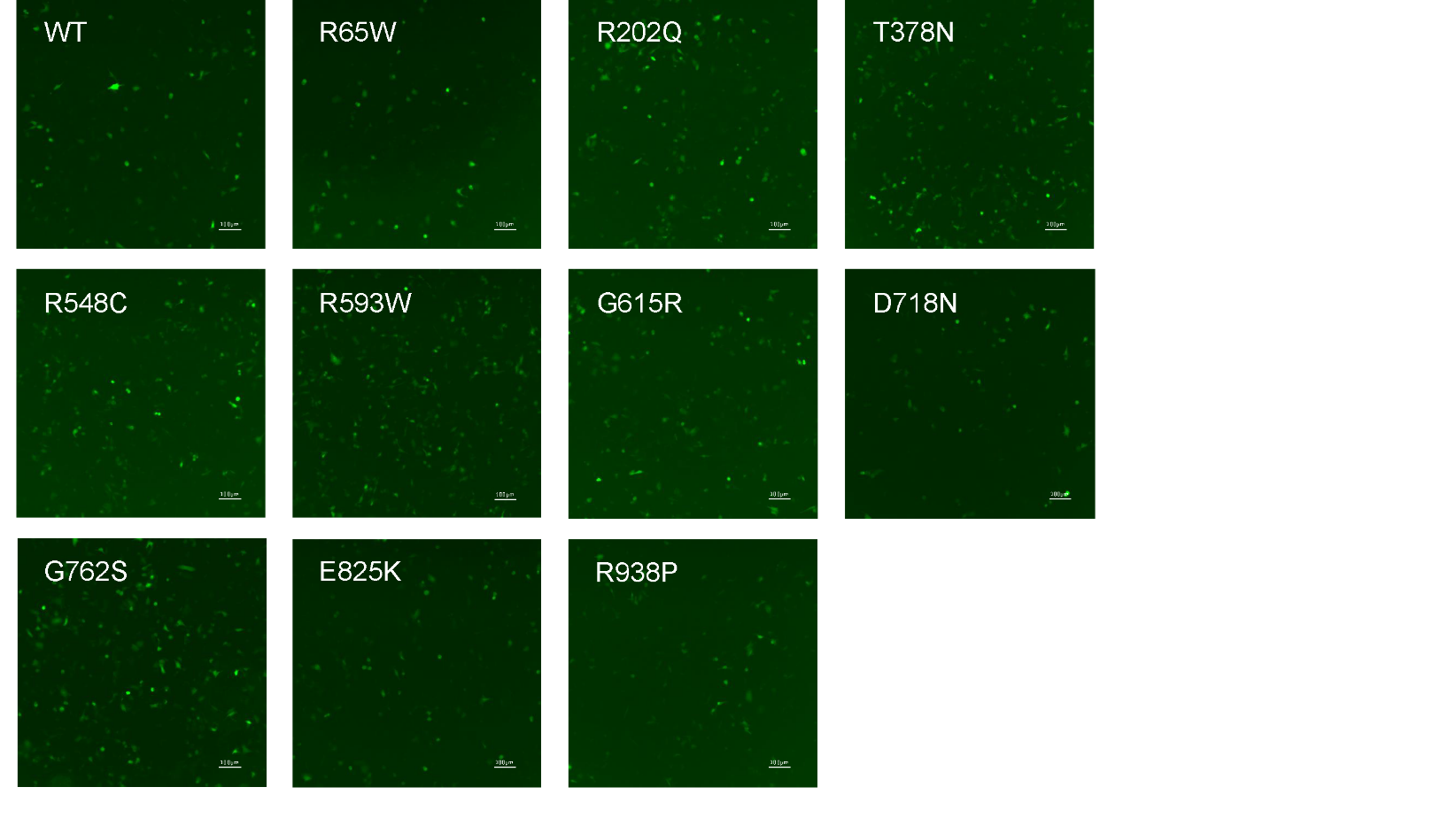


**Supplementary Figure 1.** Representative images of transfection efficiency of wild-type and mutant constructs with 100μm scales.


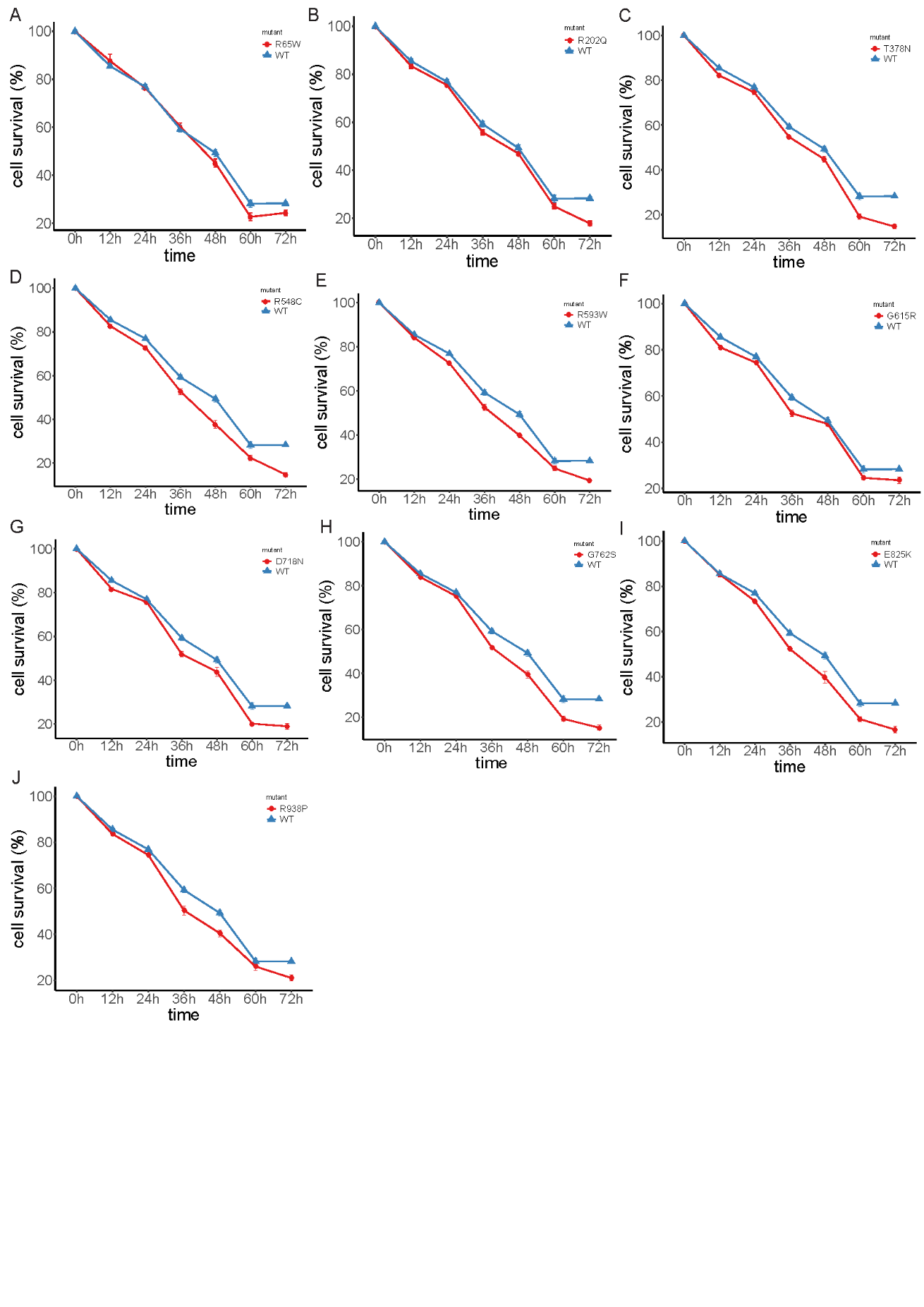


**Supplementary Figure 2.** Survival assays in HeLa cells. The survival rate of each mutant was significantly reduced compared to the wild-type (R65W: P = 0.032, other mutants: P < 0.0001, two-way ANOVA).


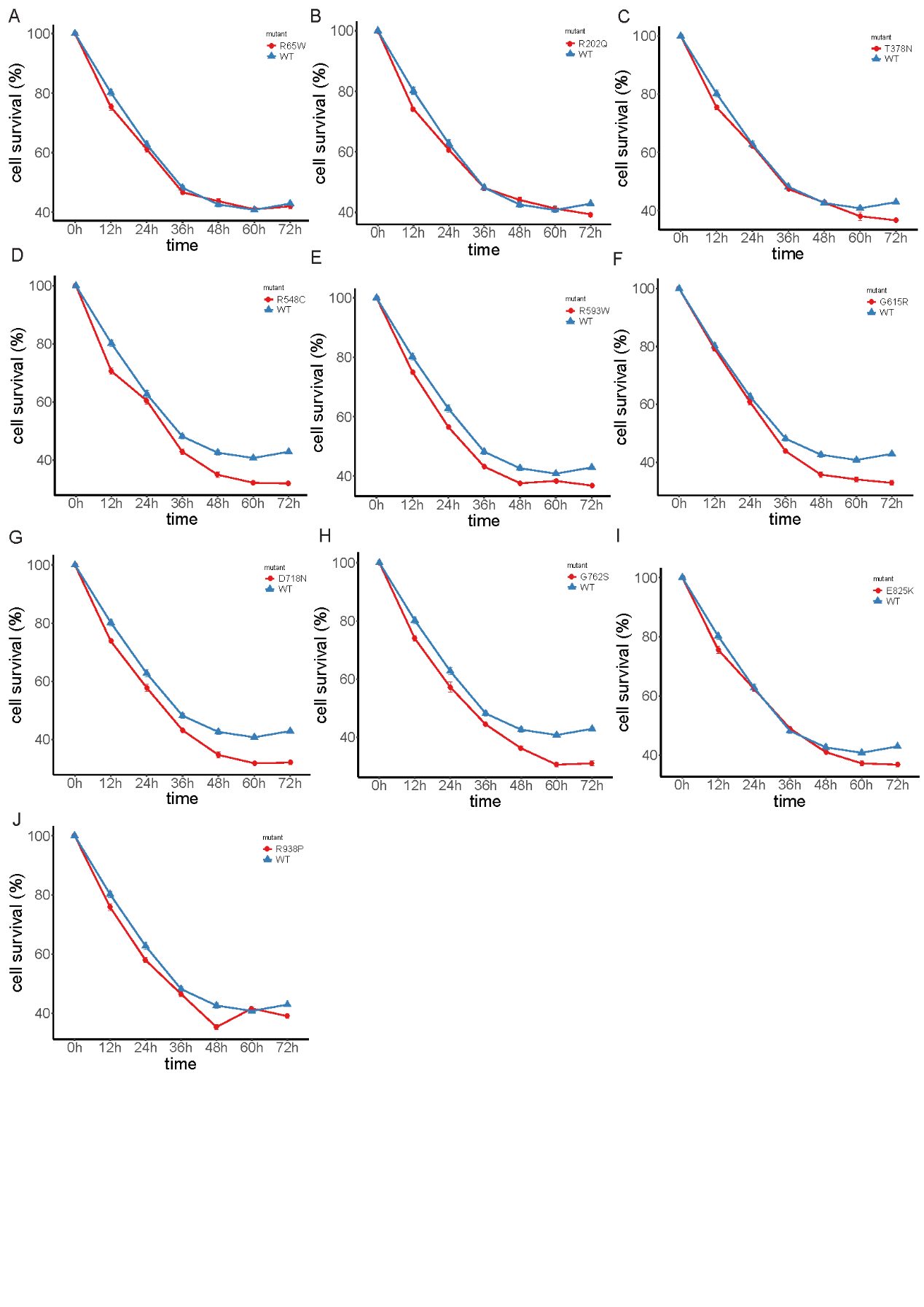


**Supplementary Figure 3.** Survival assays in HEK293T cells. The survival rate of each mutant was significantly reduced compared to the wild-type (R65W: P = 0.016, other mutants: P < 0.0001, two-way ANOVA).


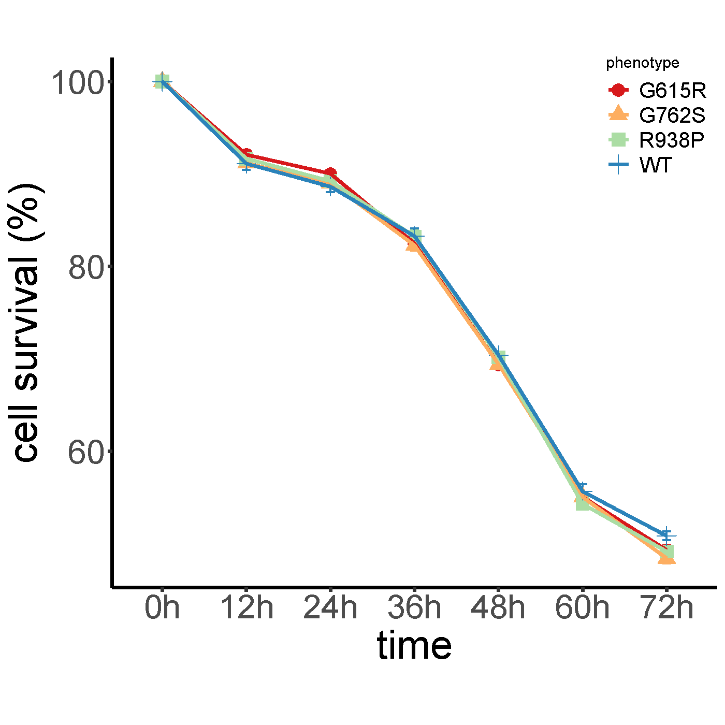


**Supplementary Figure 4** Survival assays in HeLa cells in the absence of ouabain. The survival rates of cells in each mutant group were similar to those in the WT group (P > 0.05, two-way ANOVA).

**Supplementary Table 1. Clinical characteristics of previously published patients with studied ATP1A2 mutations**

| **Mutation** | **Phenotype** | **Penetrance** | **Average age at first HM onset (years)** | **Seizure type** | **Level of intellectual disability** |
| --- | --- | --- | --- | --- | --- |
| R65W[1] | pure FHM | 3/3 | 22 | none | none |
| R202Q[2] | pure FHM (3/10 MA, 5/10 with ataxia) | 7/10 | 26 | none | none |
| T378N[3, 4] | AHC (4/4 with GTCS and mental retardation) | 4/4 | 1.9 | GTCS | mild intellectual disability |
| R548C[5] | FHM (7 members suffered from HM, 1/7 with GTCS, 5 examined, 4/5 confirmed mutations) | 4/4 | 4 | GTCS | none |
| R593W[6] | FHM (MO/MA patients not showed mutation) | 2/2 | 4 | none | none |
| G615R[7] | FHM (1/5 with generalized seizures and mental retardation; 3/5 reported childhood epilepsy, 1/3 had a learning disability) | 4/5 | 2.5 | childhood epilepsy, unknown type | mild intellectual disability |
| D718N[8] | FHM (1/7 with GTCS and delayed language development and cognitive impairment, 1/7 with low IQ) | 6/7 | 3.5 | unknown type | low IQ; mental retardation |
| G762S[9] | pure FHM | 5/5 | 21 | none | none |
| E825K[10] | FHM (1/2 with seizures) | 2/2 | 2 | febrile seizures | none |
| R938P[11] | FHM (3 with epilepsy) | NR | early childhood (infancy) | febrile seizures and GTCS | none |

FHM = familial hemiplegic migraine; MA = migraine with aura; MO = migraine without aura; AHC = alternating hemiplegia of childhood; GTCS = generalized tonic-clonic seizure; NR = not reported.

**Hemiplegic migraine pedigrees analyzed in this study.**

1. R65W[1]


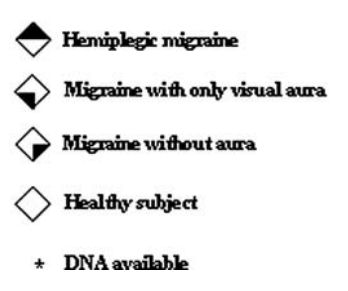

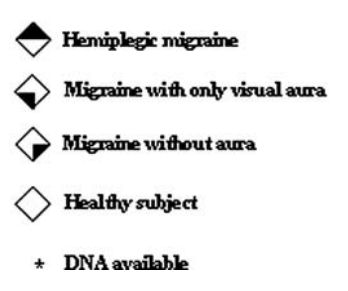

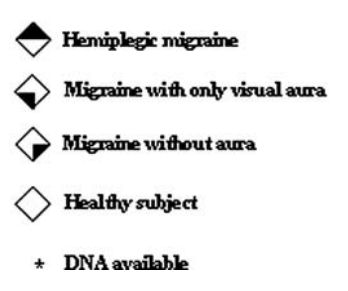


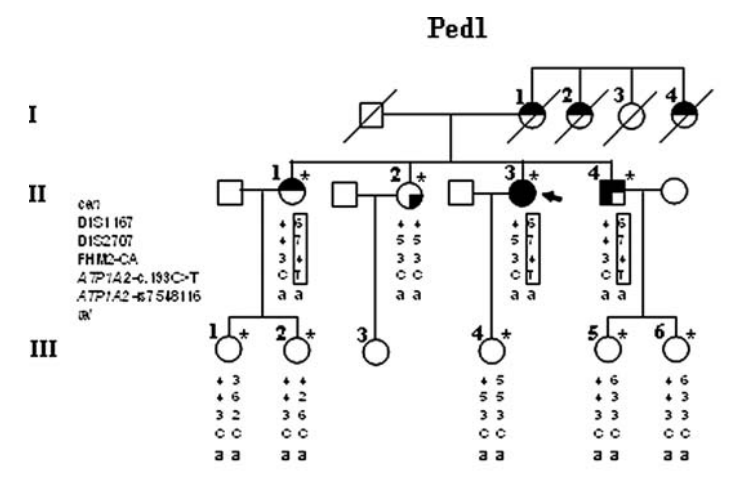


The arrows indicate the probands.

1. R202Q[2]


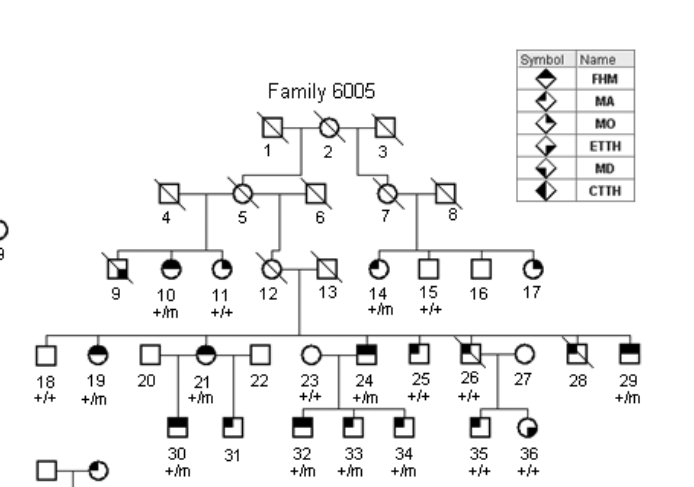


FHM: familial hemiplegic migraine; MA: migraine with aura; MO: migraine without aura; ETTH: episodic tension type headache; MD: migrainous disorder; CTTH: chronic tension type headache. FHM mutation allele is indicated with m in each family and + for wild type allele

1. T378N[3, 4]

Family A


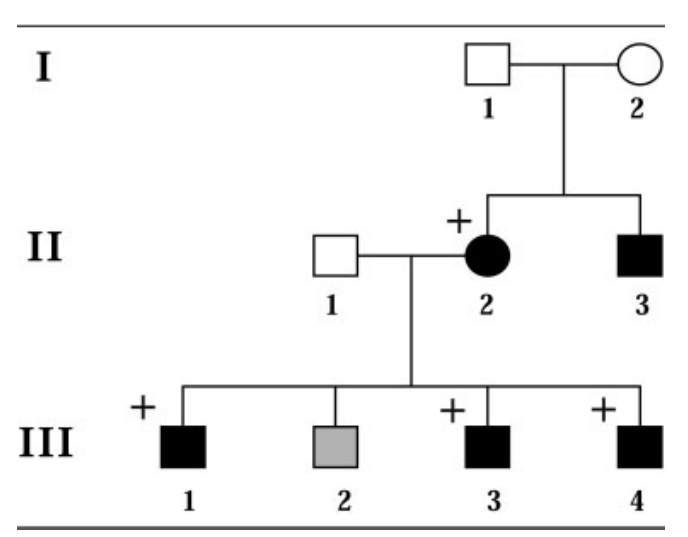


Family members with documented alternating hemiplegic/hemidystonic spells are shaded black. Plus signs in- dicate those subjects with documented generalized tonic-clonic seizures, with or without fever, and the ATP1A2 T378N mutation. Mutation analysis was not performed for II-3. Sub- ject III-2, shaded gray, likely represents a phenocopy.

Family B


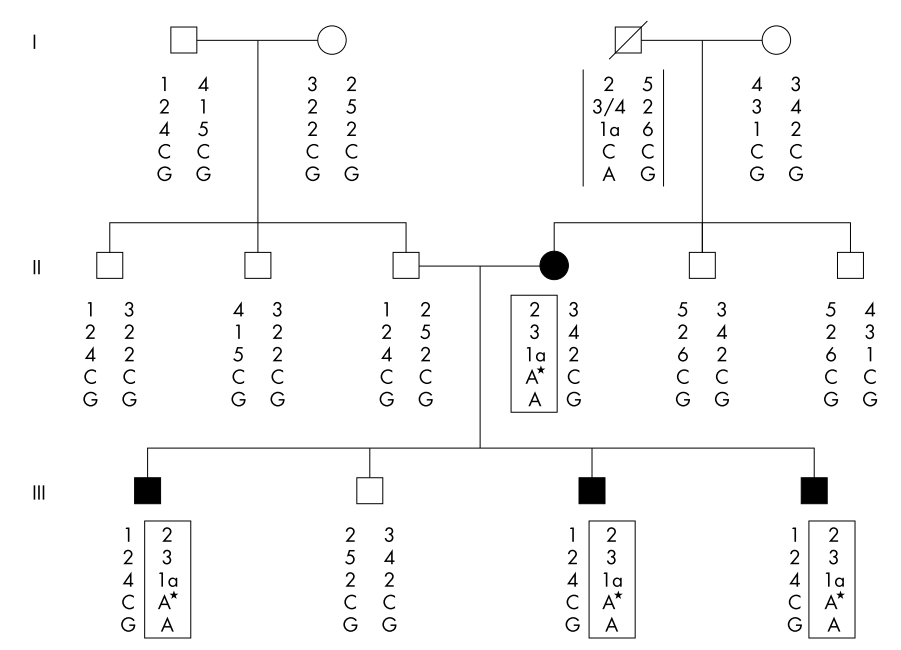


The figure shows the FHM2 locus haplotype (markers are D1S2707, NIB1152, FHM2-CA, ATP1A2/1237-C/ A*, and ATP1A2/IVS19-G/A, from top to bottom, respectively). The star indicates the base change in all affected members. Disease haplotype is boxed, while markers within open bars (individual I-3) refer to the inferred haplotype of the maternal grandfather.

1. R548C[5]


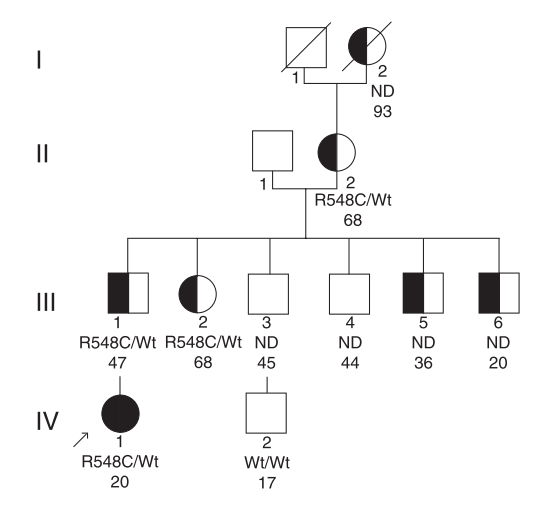


Propositus is indicated by an arrow. Symbols represent the hemiplegic migraine (left) and epilepsy (right). Genetic status and current ages are indicated. Wt, wild-type; ND, not done. Numbers above indicate current age or age at death.

1. R593W[6]


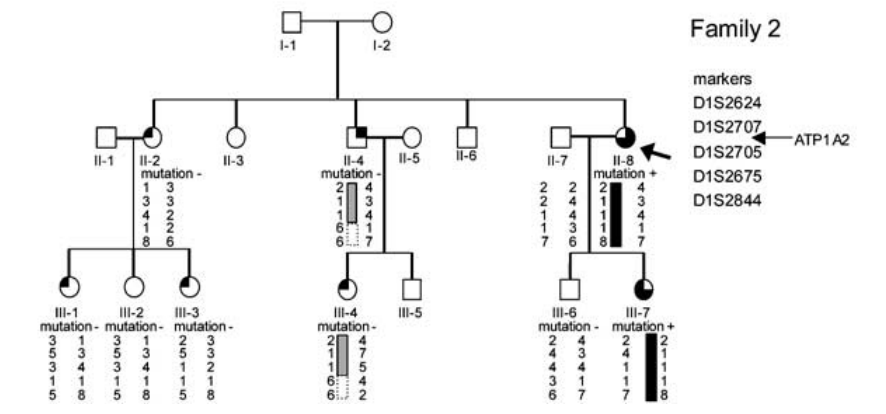


The arrows indicate the probands. The following symbols are used to indicate the diagnosis: FHM: black lower half; MA: right upper square; MO: left upper square. Mutationþ and Mutation? indicate carriers and noncarriers of the pathogenic mutations, respectively. Black bars show the haplotypes segregating with FHM; gray bars indicate the identical haplotype without the mutation; a dotted bar indicates a recombination. Genetic markers and the position of the ATP1A2 gene are indicated on the right.

1. G615R[7]


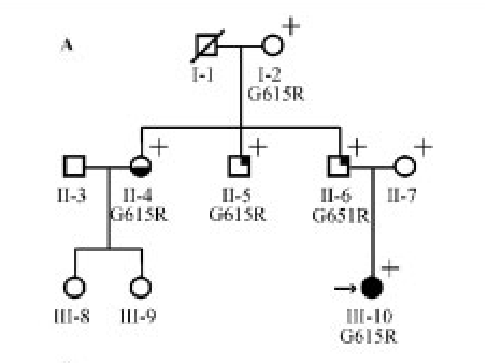


Hemiplegic migraine is indicated as a black lower halfbox (males)/ circle (females), trauma-triggered neurological episodes in the proband (arrow) as a full black circle, confusional migraine as a black upper right quadrant. A plus symbol indicates that DNA was available for genetic testing; G615R indicates het- erozygosity for the G615R mutation.

1. D718N[8]


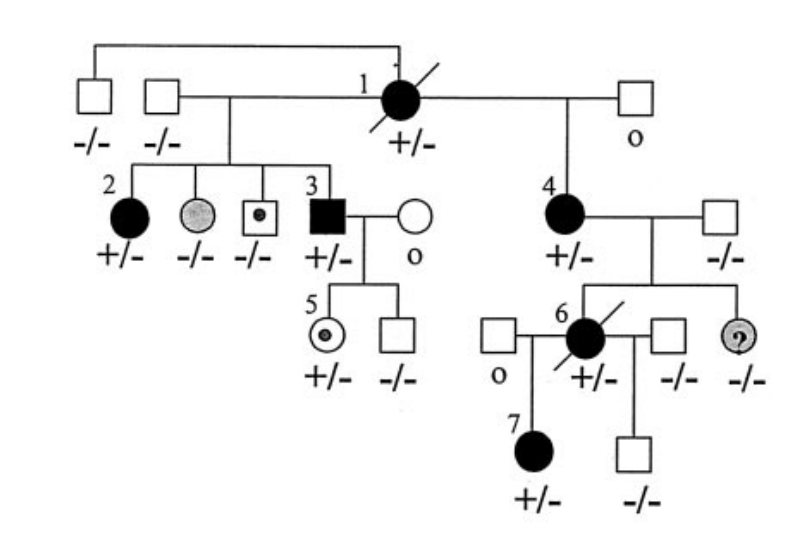


Pedigrees of the familial hemiplegic migraine (FHM) and sporadic HM cases in which ATP1A2 mutations were identified. Squares ⫽ males; circles ⫽ females; shaded symbols ⫽ individuals with different types of migraine (black ⫽ FHM; gray ⫽ migraine with aura [MA]; dot ⫽ migraine without aura; white ⫽ unaffected). Mutation carriers are num- bered for easy reference. The genotype is indicated by (⫹) for mutation carriers and by (⫺) for wild type. An (o) indicates that DNA was unavailable for testing. Note the two cases of incomplete penetrance, A5 and D2, both women ages 22 to 23. The individual with (?) in Family A was not available for the structured interview, and therefore the MA phenotype is not directly confirmed by the authors.

1. G762S[9]


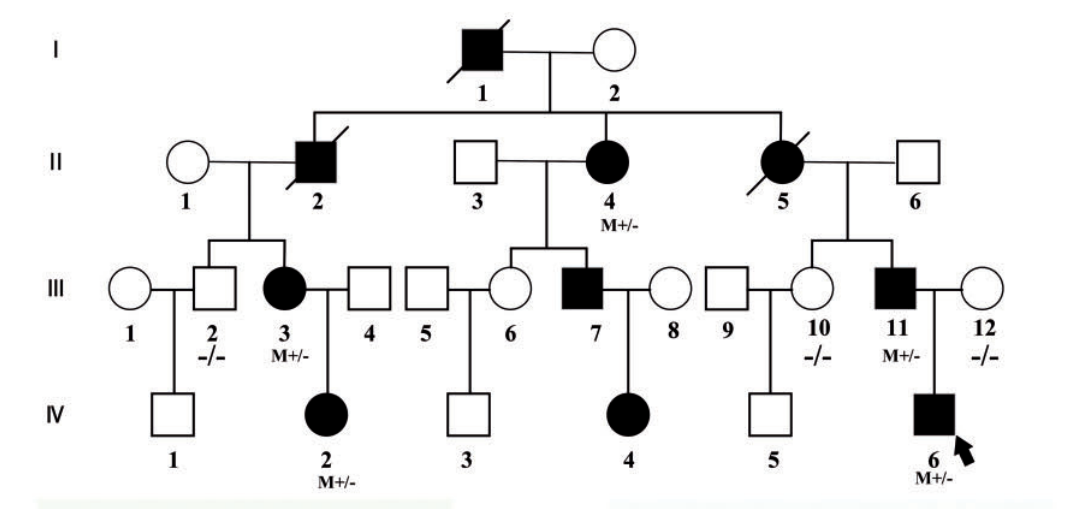


Pedigree of the family with autosomal dominant condition. Black shading indicates the affected HM patients. The black arrow shows the proband. Diagonal line: Deceased patient. M: mutation. Genotype was marked under the family members who were under gene sequencing tests.

1. E825K[10]


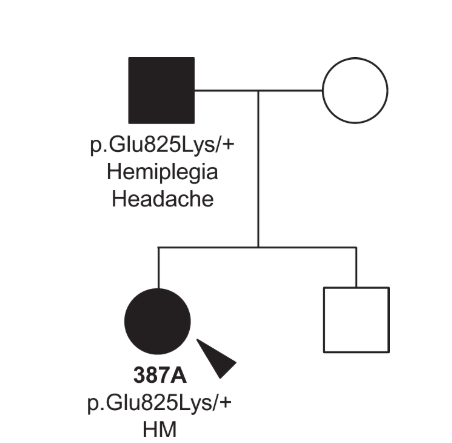


Affected individuals are denoted by solid symbols; hemiplegic migraine (HM) in black and other phenotypes in gray; squares indicate males and circles females. Probands are indicated by a black arrow. Clinical characteristics are indicated below each individual (HM, migraine with hemiplegic aura; MA, migraine with aura; CA, cerebellar atrophy; EA2, episodic ataxia type 2). Gene variant carrier status is indicated below each patient when known. Mutation p.Ser218Leu appeared de novo in the affected sib.

1. R938P[11]

A 15-year-old girl presented with a 3-day history of headache, photophobia, nausea, vomiting, and dizziness. She became disorientated, drowsy, and eventually unresponsive and incontinent.

On arrival to the emergency department she was self-ventilating and Glasgow Coma Scale was 9/15. She was pyrexic with a temperature of 38.98C. There was a left facial droop. She moved her right side spontaneously but not her left side. Reflexes were brisk on the left side and the left plantar was extensor.

Electroencephalogram study was performed. She was treated empirically with intravenous ceftriaxone and aciclovir along with supportive care. Her level of consciousness improved over 48 hours and a dense left hemiparesis became evident. On day 3 and 4 of her admission, she had a series of complex partial seizures with unresponsiveness, left face and arm twitching. She was treated with intravenous lorazepam, phenytoin, and levetir- acetam. She received intensive inpatient rehabilitation, and the hemiparesis resolved over 4 weeks.

Her medical history included epilepsy in early childhood (febrile seizures in infancy followed by unprovoked generalized tonic-clonic seizures until age 5). At age 12 there was an episode of numbness and weakness in the left leg lasting 2 hours. Her father had febrile convulsions in childhood and her paternal uncle has epilepsy. Her paternal grandmother has symptoms of hemiplegic migraine.

**References**

1. Tonelli A, Gallanti A, Bersano A, et al (2007) Amino acid changes in the amino terminus of the Na,K-adenosine triphosphatase alpha-2 subunit associated to familial and sporadic hemiplegic migraine. Clin Genet 72:517–523. https://doi.org/10.1111/j.1399-0004.2007.00892.x

2. Thomsen LL, Kirchmann M, Bjornsson A, et al (2007) The genetic spectrum of a population-based sample of familial hemiplegic migraine. Brain 130:346–356. https://doi.org/10.1093/brain/awl334

3. Swoboda KJ, Kanavakis E, Xaidara A, et al (2004) Alternating hemiplegia of childhood or familial hemiplegic migraine?: A novel ATP1A2 mutation. Ann Neurol 55:884–887. https://doi.org/10.1002/ana.20134

4. Bassi MT, Bresolin N, Tonelli A, et al (2004) A novel mutation in the ATP1A2 genes causes alternating hemiplegia of childhood. J Med Genet 41:621–628. https://doi.org/10.1136/jmg.2003.017863

5. Lebas A, Guyant-Maréchal L, Hannequin D, et al (2008) Severe attacks of familial hemiplegic migraine, childhood epilepsy and ATP1A2 mutation. Cephalalgia 28:774–777. https://doi.org/10.1111/j.1468-2982.2008.01603.x

6. Vanmolkot KRJ, Kors EE, Turk U, et al (2006) Two de novo mutations in the Na,K-ATPase gene ATP1A2 associated with pure familial hemiplegic migraine. Eur J Hum Genet 14:555–560. https://doi.org/10.1038/sj.ejhg.5201607

7. Vanmolkot KRJ, Stroink H, Koenderink JB, et al (2006) Severe episodic neurological deficits and permanent mental retardation in a child with a novel FHM2 ATP1A2 mutation. Ann Neurol 59:310–314. https://doi.org/10.1002/ana.20760

8. Jurkat-Rott K, Freilinger T, Dreier JP, et al (2004) Variability of familial hemiplegic migraine with novel A1A2 Na +/K+-ATPase variants. Neurology 62:1857–1861. https://doi.org/10.1212/01.WNL.0000127310.11526.FD

9. Tang W, Zhang M, Qiu E, et al (2019) A Chinese family with familial hemiplegic migraine type 2 due to a novel missense mutation in ATP1A2. Cephalalgia 39:1382–1395. https://doi.org/10.1177/0333102419847738

10. Carreño O, Corominas R, Angèlica Serra S, et al (2013) Screening of CACNA1A and ATP1A2 genes in hemiplegic migraine: Clinical, genetic, and functional studies. Mol Genet Genomic Med 1:206–222. https://doi.org/10.1002/mgg3.24

11. Murphy OC, Merwick A, O’Mahony O, et al (2018) Familial Hemiplegic Migraine with Asymmetric Encephalopathy Secondary to ATP1A2 Mutation: A Case Series. J Clin Neurophysiol 35:e3–e7. https://doi.org/10.1097/WNP.0000000000000387
